# Hypoventilation Syndrome Secondary to Club-Shaped Chest Wall Deformity

**DOI:** 10.7759/cureus.19785

**Published:** 2021-11-21

**Authors:** Abdullah M Almazloum, Faaezuddin Syed, Safwan U Abbasi, Sameh Shalaby, Sami Almustanyir

**Affiliations:** 1 Pulmonology Department, Prince Mohamed Bin Abdulaziz Hospital, Riyadh, SAU; 2 College of Medicine, Alfaisal University, Riyadh, SAU

**Keywords:** club shaped chest, hypoxemia, hypercapnia, alveolar hypoventilation, hypoventilation syndrome, chest wall deformity

## Abstract

Hypoventilation syndrome is defined as a decrease in alveolar ventilation leading to hypercapnia (PaCO_2 _> 35-45 mmHg) and hypoxemia. There are multiple causes of hypoventilation syndrome described in the literature, of which central and obesity-related causes are more prevalent. Other causes such as neuromuscular disorders and chest wall deformities are relatively less common. Multiple defects in the normal functioning of the respiratory function are implicated in the pathophysiological mechanism of hypoventilation syndrome, such as a hypoactive central ventilatory drive, decreased airway function, ventilation-perfusion mismatch, defective pulmonary mechanics, and respiratory muscle fatigue. Patients often present with dyspnea, headache, lethargy, repeated pulmonary infections, hypoxia that usually improves with low flow oxygen, and hypercapnia that may alter mental function. Nocturnal or diurnal assisted mechanical ventilation is proven to be an effective therapy for patients suffering hypoventilation syndromes. We describe a case of a 47-year-old woman with hypoventilation syndrome resulting from a rare chest wall deformity with inward protrusion of the costochondral junction of the ribs with ossification of the costal cartilage on CT who presented with dyspnea and hypercapnia.

## Introduction

Alveolar hypoventilation is defined as a decrease in ventilation leading to hypercapnia, which, on arterial blood gas (ABG) analysis, manifests as an increase in partial pressure of carbon dioxide (PaCo_2_), beyond the normal range of 35-45mmHg [1]. Hypercapnia alongside hypoxemia (low partial pressure of oxygen or PaO_2_), seen in hypoventilating patients, accounts for the clinical manifestations of hypoventilation syndrome [2,3]. Patients often present with dyspnea, somnolence, headache, lethargy, repeated pulmonary infections, and altered mental function [1,2]. Several mechanisms have been illustrated in the literature for the acute and chronic causes of alveolar hypoventilation and hypoventilation syndromes, of which central and obesity (obesity hypoventilation syndrome or OHS) related causes are elucidated in detail. Remainder of the causes of hypoventilation are related to chest wall deformities and neuromuscular disorders. The causes of hypercapnia in these patients are various and not yet completely known; however, the basic etiology points at impaired respiratory dynamics [2,3].

## Case presentation

We report a case of a 47-year-old married woman from rural Saudi Arabia. She is of short stature and a known case of chronic obstructive pulmonary disease (COPD) on PRN Salbutamol MDI; she presented for evaluation of dyspnea after an upper respiratory tract infection (URTI). The dyspnea was worsened on lying down and walking short distances and was also accompanied by occasional hemoptysis.

This was the first time that the patient was admitted for any pulmonary complaints. The patient’s past medical history is significant for acute hepatitis A in childhood. She is sterile with no children. She does not smoke or suffer from any allergies nor has any significant environmental exposure. She was afebrile when admitted, with other vitals being within normal limits, except for her oxygen saturation which was 93% on room air. She was conscious, alert, and oriented. Her height was recorded as 142 cm and her weight was 53 kg (BMI = 26 kg/m^2^). There was no cough or dyspnea present at the time of clinical examination. Chest auscultation revealed bilateral basal crackles, and the extremities examination was significant for bilateral lower limb edema. Rest of the physical examination was unremarkable.

**Figure 1:**
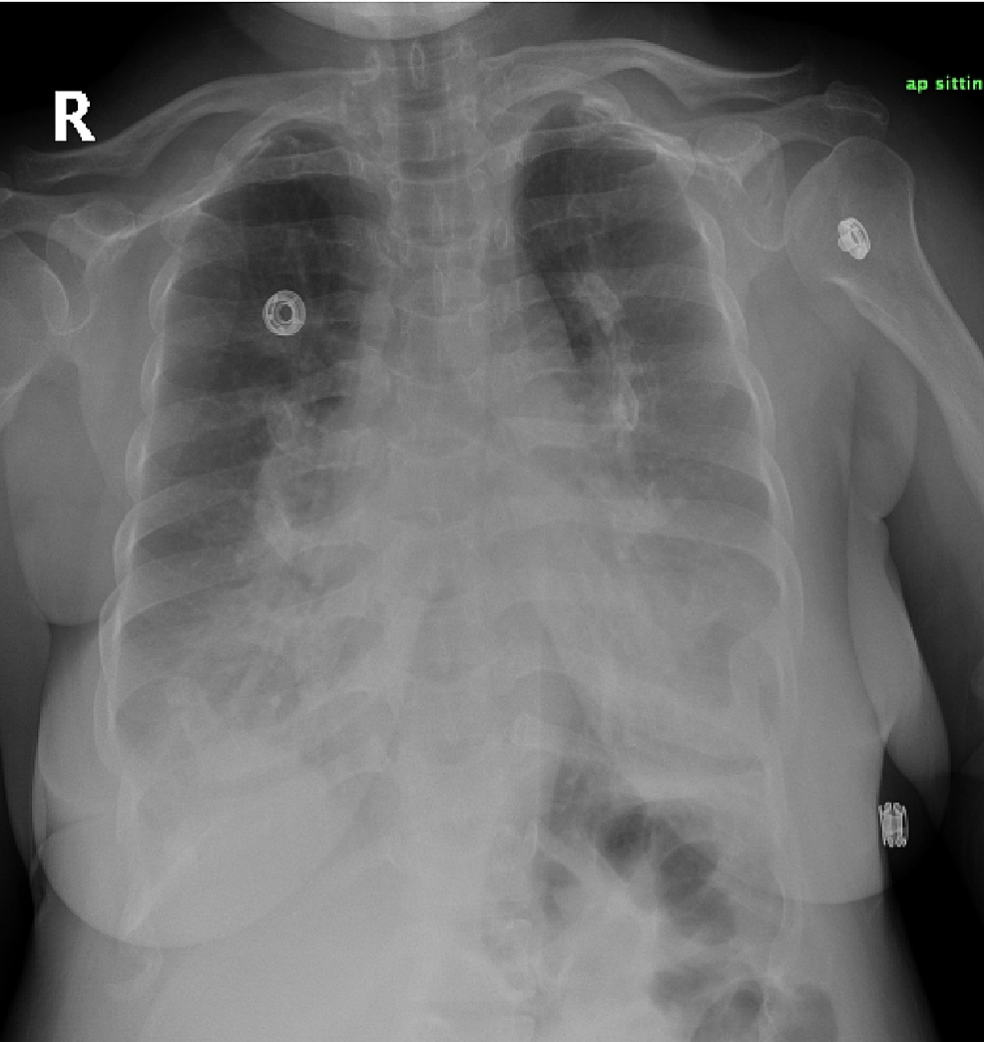
Enlarged cardiac shadow with hazy heart boundaries, bilaterally obliterated cardiophrenic and costophrenic angles, lower zones opacified in comparison to upper zones are seen. These findings are suggestive of alveolar airspace disease with bilateral pleural effusion, consistent with diagnosis of congestive heart failure. However, the CT study revealed something different. CT, computed tomography

**Figure 2:**
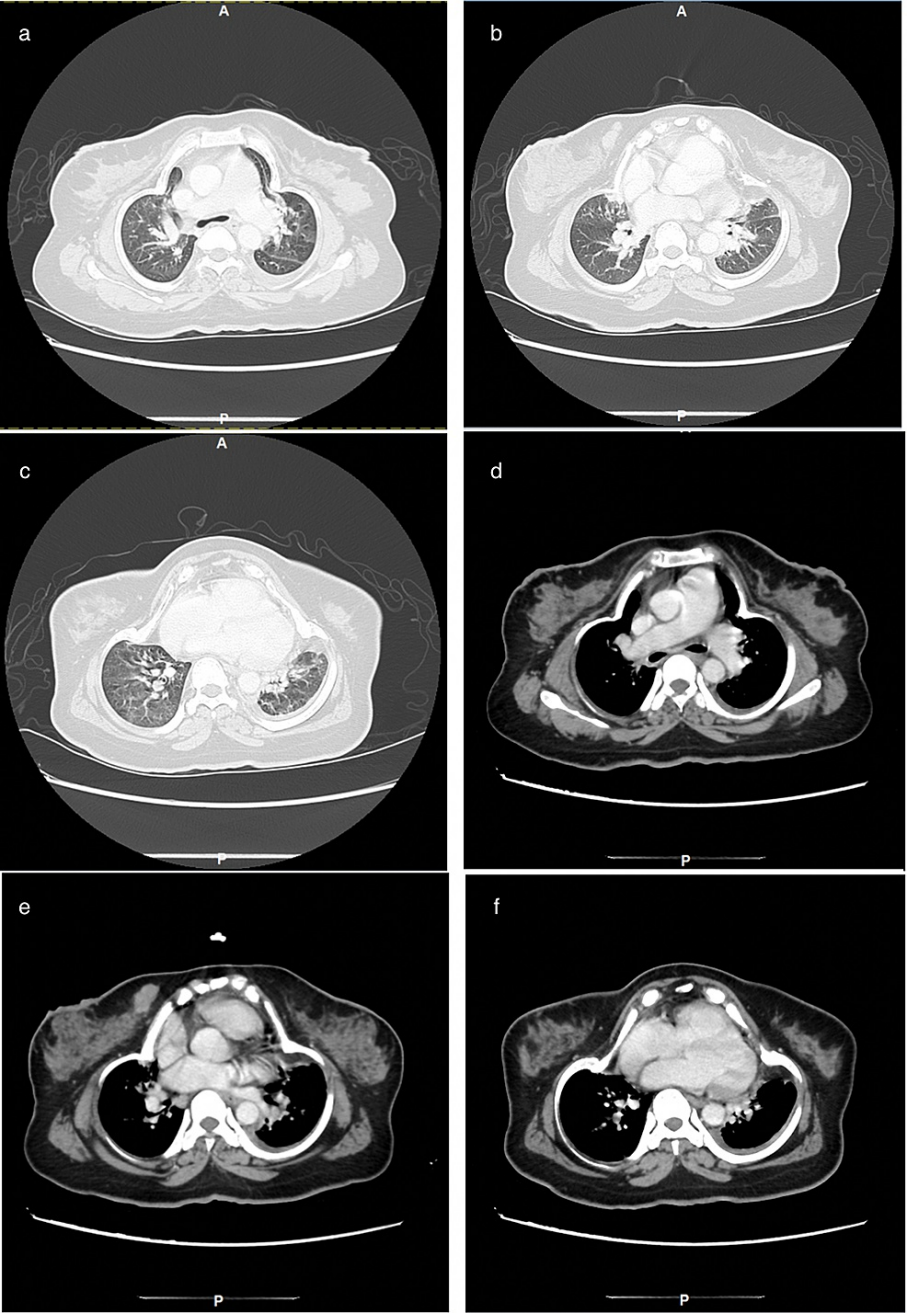
Lung and mediastinal window in axial view shows deformed chest wall with lungs and heart taking its club-like shape. No apparent parenchymal lesions apart from a rim of left-sided effusion are seen.

**Figure 3:**
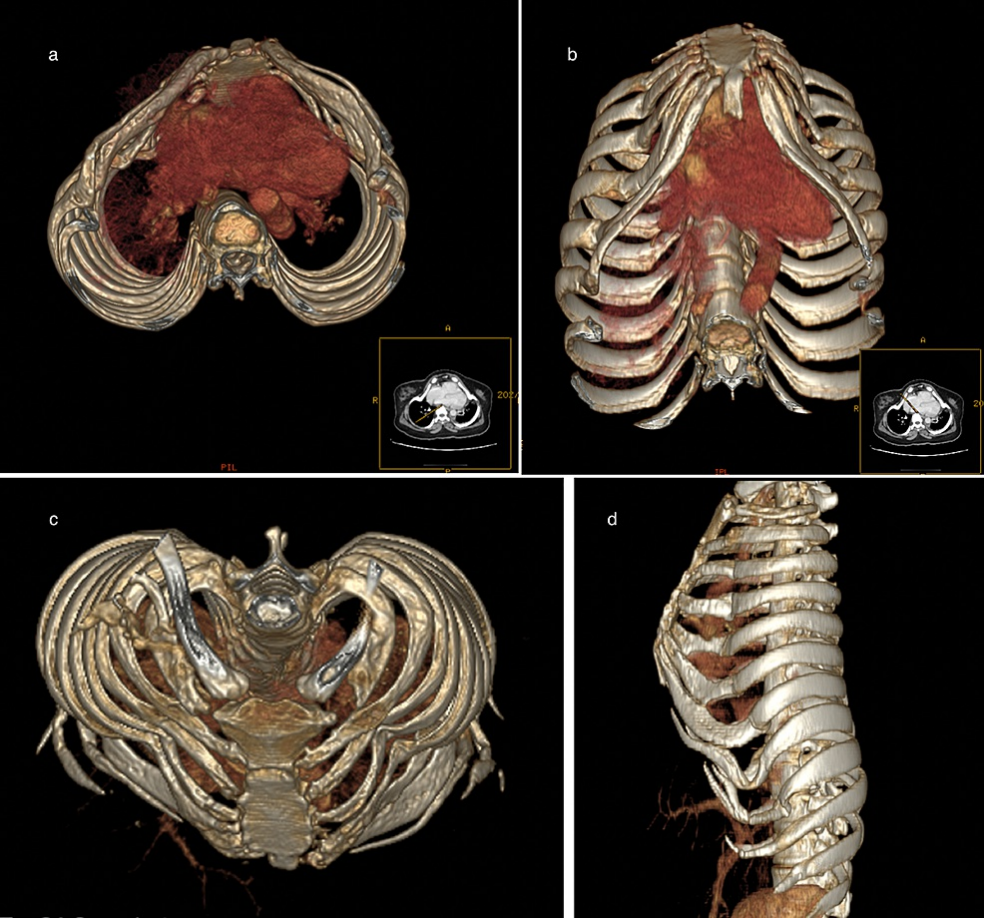
Three-dimensional reconstruction images of different views.

ABG was significant for hypercapnia with pH of 7.36, PaCO_2_ of 71mmHg, PaO_2_ of 52mmHg, and HCO_3_ of 35mEq/L. An abdominal ultrasound was performed, which showed mildly coarse liver parenchyma, a contracted gallbladder containing an isoechoic structure adherent to the wall measuring 0.3 cm by 0.2 cm, and minimal left-sided pleural effusion. Rest of the abdominal ultrasound was insignificant. Chest X-ray showed bilateral obliteration of the costophrenic angle and bilateral lower zone consolidations, finding that can be (misguidedly) interpreted as bilateral pneumonia with effusions (Figure 1). Echocardiogram was performed and was unremarkable with an ejection fraction (EF) of 55%. A chest CT with contrast was also ordered and it showed inward protrusion of the costochondral junction of the ribs with ossification of the costal cartilage leading to a narrowed thoracic cavity. A deformity of the shape of the heart was also noticed due to the findings stated earlier. Apart from minimal left-sided pleural effusion and small-sized (albeit clear) lungs, rest of the chest CT was unremarkable with no evidence of parenchymal or airways abnormalities (Figure 2; three-dimensional reconstruction images of different views can be seen in Figure 3). Hence, a diagnosis of hypoventilation syndrome secondary to chest wall deformity was made. She was administered a diuretic based on impression of cardiomegaly with bilateral pleural effusion seen on initial chest X-ray, suggestive of decompensated heart failure. Unfortunately, she has not been followed up with since owing to her residence being in a rural area.

## Discussion

Hypercapnia with the evidence of hypoxemia on ABG is an indicator of an underlying hypoventilation syndrome. Various causes of hypoventilation syndrome have been explained in the literature, mainly constituting of central alveolar hypoventilation, OHS (BMI > 30kg/m^2^ with no other identifiable cause of daytime hypercapnia), chest wall deformities, neuromuscular disorders, parenchymal lung diseases, and obstructive lung diseases [1,3,4]. Diverse aberrations in the normal functioning of the respiratory system have been identified as a cause of hypoventilation syndrome, which may result from a hypoactive central ventilatory drive, decreased airway function, ventilation-perfusion mismatch, defective pulmonary mechanics, and respiratory muscles fatigue [2].

Chest wall deformities can occur as a result of trauma, surgical procedure, or neuromuscular disease, can result secondary to diseases affecting the spine, connective tissue disorders, or congenital anomalies, or can be idiopathic. Kyphoscoliosis affects majority of the patients presenting with hypoventilation syndrome as a consequence of chest wall deformity [1,5]. The reduction in chest wall compliance leads to a reduced tidal volume (VT), which contributes to the hypoventilation. Additionally, the pulmonary vasculature may be deranged in such patients, with factors such as mechanical compression of the vasculature and medial hypertrophy of the vessels contributing to the overall symptomatology of the resultant hypoventilation syndrome [5].

Even though the episodes of hypoventilation can occur at any time of the day, patients are often more susceptible to experience an episode during sleep. During the rapid eye movement (REM) stage of the sleep cycle, there is a decline in muscles’ tone, the relevant ones of which are the intercostal muscles, which become atonic. This leads to the work of breathing to be primarily carried out by the diaphragm. Patients suffering from muscle disorders or having weak respiratory muscles or patients with chest wall disorders are often more susceptible to hypoxemia and hypercapnia, as a drop in muscle tone often predisposes them to reduced airflow, a decline in chest wall movement, and eventually alveolar hypoventilation [1,2,6].

With a BMI of less than 30 kg/m^2^, no identifiable central cause, and no clinical features of neuromuscular disorders, a majority of the causes of hypoventilation syndrome can safely be ruled out in our patient. Hypoxemia on pulse oximetry and ABG with dyspnea alongside evidence of hypercapnia points to a hypoventilation syndrome. Radiological findings such as inward protrusion of the costochondral junction with ossification of the costal cartilage and a narrowed thoracic cavity diagnosed our patient to be a case of hypoventilation syndrome secondary to a chest wall deformity. The shape of the chest deformity seen in the axial CT images of our patient is somewhat rare, which looks like a “club ♣ shape,” where the upper part of the club contains the heart and the great vessels without adjacent lungs on either side.

She was misguidedly labelled to have COPD due to retaining of CO_2_ on ABG analysis, for which she was prescribed bronchodilators.

Assisted non-invasive mechanical ventilation at night time (in some cases during daytime) has been recommended as a mode of therapy for patients suffering from hypoventilation disorders. It has been shown to moderately improve the symptoms as well as the underlying mechanics, with effects lasting throughout the day and even at night [2]. This is done mainly by correcting the chest wall compliance, returning the chemoreceptor function to normal, and providing rest to the muscles involved in respiration. Patients have been shown to witness fewer symptoms of hypoventilation when assisted through nocturnal mechanical ventilation [6]. Studies have also shown a decline in the mortality rate in patients with chronic use of nocturnal positive pressure ventilation [1].

## Conclusions

Chest wall deformity is an established cause of hypoventilation syndrome. It is quite common in medical practice to find a mistaken diagnosis of hypoventilation syndrome as COPD based on unexplained etiology of hypercapnia. However, definitive diagnosis requires relevant blood tests to assess for hypercapnia and hypoxemia, in addition to proper radiological imaging and pulmonary function testing to evaluate for the underlying etiology of hypercapnia. Prompt diagnosis and effective treatment are necessary to alleviate the signs and symptoms and inevitably improve the patients’ quality of life.
